# Experimental Comparison of Knife-Edge and Multi-Parallel Slit Collimators for Prompt Gamma Imaging of Proton Pencil Beams

**DOI:** 10.3389/fonc.2016.00156

**Published:** 2016-06-27

**Authors:** Julien Smeets, Frauke Roellinghoff, Guillaume Janssens, Irene Perali, Andrea Celani, Carlo Fiorini, Nicolas Freud, Etienne Testa, Damien Prieels

**Affiliations:** ^1^Ion Beam Applications SA, Louvain-la-Neuve, Belgium; ^2^IPNL, Université Lyon 1 and CNRS/IN2P3, Lyon, France; ^3^Université Lyon, INSA – Lyon, Université Lyon 1, UJM-Saint Etienne, CNRS, Inserm, Centre Léon Bérard, CREATIS UMR 5220 U1206, Lyon, France; ^4^Politecnico di Milano, Dipartimento di Elettronica, Informazione e Bioingegneria, Milano, Italy; ^5^Istituto Nazionale di Fisica Nucleare, Sezione di Milano, Milano, Italy; ^6^XGLab SRL, Milano, Italy

**Keywords:** proton therapy, range verification, prompt gamma imaging

## Abstract

More and more camera concepts are being investigated to try and seize the opportunity of instantaneous range verification of proton therapy treatments offered by prompt gammas emitted along the proton tracks. Focusing on one-dimensional imaging with a passive collimator, the present study experimentally compared in combination with the first, clinically compatible, dedicated camera device the performances of instances of the two main options: a knife-edge slit (KES) and a multi-parallel slit (MPS) design. These two options were experimentally assessed in this specific context as they were previously demonstrated through analytical and numerical studies to allow similar performances in terms of Bragg peak retrieval precision and spatial resolution in a general context. Both collimators were prototyped according to the conclusions of Monte Carlo optimization studies under constraints of equal weight (40 mm tungsten alloy equivalent thickness) and of the specificities of the camera device under consideration (in particular 4 mm segmentation along beam axis and no time-of-flight discrimination, both of which less favorable to the MPS performance than to the KES one). Acquisitions of proton pencil beams of 100, 160, and 230 MeV in a PMMA target revealed that, in order to reach a given level of statistical precision on Bragg peak depth retrieval, the KES collimator requires only half the dose the present MPS collimator needs, making the KES collimator a preferred option for a compact camera device aimed at imaging only the Bragg peak position. On the other hand, the present MPS collimator proves more effective at retrieving the entrance of the beam in the target in the context of an extended camera device aimed at imaging the whole proton track within the patient.

## Introduction

Proton therapy materializes the medical physicist’s goal to specifically target tumor volumes while sparing surrounding healthy – and potentially critical – organs. But this improved precision demands improved accuracy in order to prevent any under- or overshoot. Safety margins are applied, and research efforts are invested in order to reduce range uncertainties before treatment delivery, monitor range during treatment, and verify range after treatment. Luckily, proton therapy offers several distinctive opportunities for treatment quality control, for example through activated nuclei along the proton beam path that can be imaged by a PET scan device (1), through proton-induced acoustic waves that could be measured by an ultra-sound probe (2), or through physiological impacts that can later be observed on MRI acquisitions (3).

In this regard, Jongen and Stichelbaut (4) suggested to image the prompt gammas emitted by proton-excited nuclei in order to take advantage of the straightforward correlation of their spatial emission distribution with the proton range. First experimental evidences reported by Min et al. (5) triggered interest for the Prompt Gamma Imaging (PGI) approach and its promises of instantaneous feedback on an individual spot basis with non-invasive equipment. Recent efforts culminated in the alternative ideas of taking benefit from the time emission distribution of prompt gammas through the Prompt Gamma Timing (PGT) method by Golnik et al. (6), or from their energy emission distribution through the Prompt Gamma Spectroscopy (PGS) method by Verburg and Seco (7).

The PGI, PGT, and PGS approaches have their own specificities, advantages, and disadvantages in terms of sensitivity, generated information, cost, footprint, supported beam conditions, and robustness to different sources of uncertainties. The preferred approach is thereby dependent on the favored features. In the near future, the ongoing development of prototype systems will hopefully allow experimental comparisons in order to assess what approach is offering the preferred tradeoff depending on the clinical context under consideration (clinical case, treatment mode, treatment workflow).

The PGI field has been very dynamic over the last years, with a large number of camera concepts being investigated, optimized, and prototyped. These are not only relying on passive collimators but also on sophisticated electronic collimation techniques through different designs of Compton cameras (8) that offer the advantage of discarding the negative impact of a passive collimator in terms of weight and signal attenuation, at the cost of reduced scoring efficiency in the detection stages and increased complexity in electronics and data treatment.

The present study is focused on PGI, more specifically with passive collimators, in order to leverage on the promises of this option as the one most suited to diagnosing the largest number of spots of a pencil beam scanning (PBS) treatment delivery and the one most accommodating the various beam time structures of the different types of accelerators used in clinical facilities (synchrotron, cyclotron, and synchrocyclotron), the maximum instantaneous clinical beam currents of which can differ over several orders of magnitude.

The information on which feedback is presently missing during treatment delivery is the beam penetration depth within the patient, so that 1D imaging was most often privileged so far, with two main options in the form of multi-parallel slit (MPS) (9) and knife-edge slit (KES) (10) collimators meant at producing the best possible projection of the prompt gamma emission fall-off ~3 mm before the proton mean maximum penetration depth. The concrete, practical objective of the present study is the experimental comparison of the performance of these two types of collimators in combination with the first prompt gamma camera prototype built by Perali et al. (11) in order to identify the most advantageous design for the clinical evaluation of the camera prototype. In addition to the Bragg peak position, the performance of both collimators in retrieving the entrance point of the beam in the target is also compared. In case of absence of complementary imaging modalities, the measurement of the entrance point could help diagnose the cause of any mismatch of the Bragg peak position with respect to treatment plan expectations.

## Materials and Methods

### Camera Design

Individual spots of a PBS treatment plan typically range between 10^6^ and 10^8^ protons for a typical 2 Gy fraction. Nuclear collisions cause ~1 prompt gamma to escape the patient every 10 protons (12), resulting in a large number of prompt gammas per spot. But these are challenging to detect as they are emitted instantaneously, with multi-MeV energy and spread over 4π steradians. The use of a thick collimator as well as fast and dense crystals is therefore required, which in turn limits the solid angle that can be covered by a camera device of reasonable weight and cost. As a consequence, the spatial resolution of the collimator needs to be compromised in order to favor the counting statistics and achieve a clinically viable efficiency of the order of 1 prompt gamma detected every 10^5^ protons (13). The subsequent, poor spatial resolution as well as the significant statistical fluctuations of the signal of a single spot is then compensated for by the use of *a priori* information when comparing the actually measured profile to a reference computed one reflecting the treatment plan hypotheses (14).

The present study relies on the first unit prompt gamma camera built by Perali et al. (11) that demonstrated, in combination with a KES collimator, sufficient detection speed and efficiency for compatibility with clinical irradiation scenarios. The camera is a dedicated, very-fast, 1-dimensional, high-energy gamma imaging device built upon two rows of 20 LYSO crystal slabs, directly coupled to arrays of SiPMs (Silicon Photomultipliers) and read out by 40 independent acquisition channels that can be operated in two different modes. During a proton irradiation, each channel is operated in fast mode and scores the number of events that are detected above a first, lower-threshold comparator and below a second, upper-threshold comparator. The levels of these two comparators correspond to the energy selection window of the camera. They are set as a result of the camera energy calibration, based on spectra of known energy lines acquired in slow mode. Each of the 40 LYSO slabs is 4 mm wide along beam axis, 100 mm high, and 31.5 mm deep, for a total crystal volume of 504 cm^3^ producing a 1D image of 8 cm width.

### Collimator Designs

Two collimators made of tungsten alloy (17.0 g/cm^3^) were prototyped for experimental comparison. The first one is a KES collimator design reproducing dimensions from Smeets et al. (12) that were selected by eye inspection of the detection profiles resulting from extensive parameter variation tests with Monte Carlo code MCNPX version 2.5.0 (15). The second one is a MPS collimator design implementing the conclusions of Roellinghoff (16) for an optimal prompt gamma profile falloff retrieval precision from extensive parameter variation tests with simulation platform GATE version 6 (17) built upon Monte Carlo code Geant4 version 9.4p01, under the first constraint of the 4 mm segmentation of the present camera system and the second constraint of a weight equal to that of the walls of the 40 mm thick KES collimator for direct comparability.

**Figure 1:**
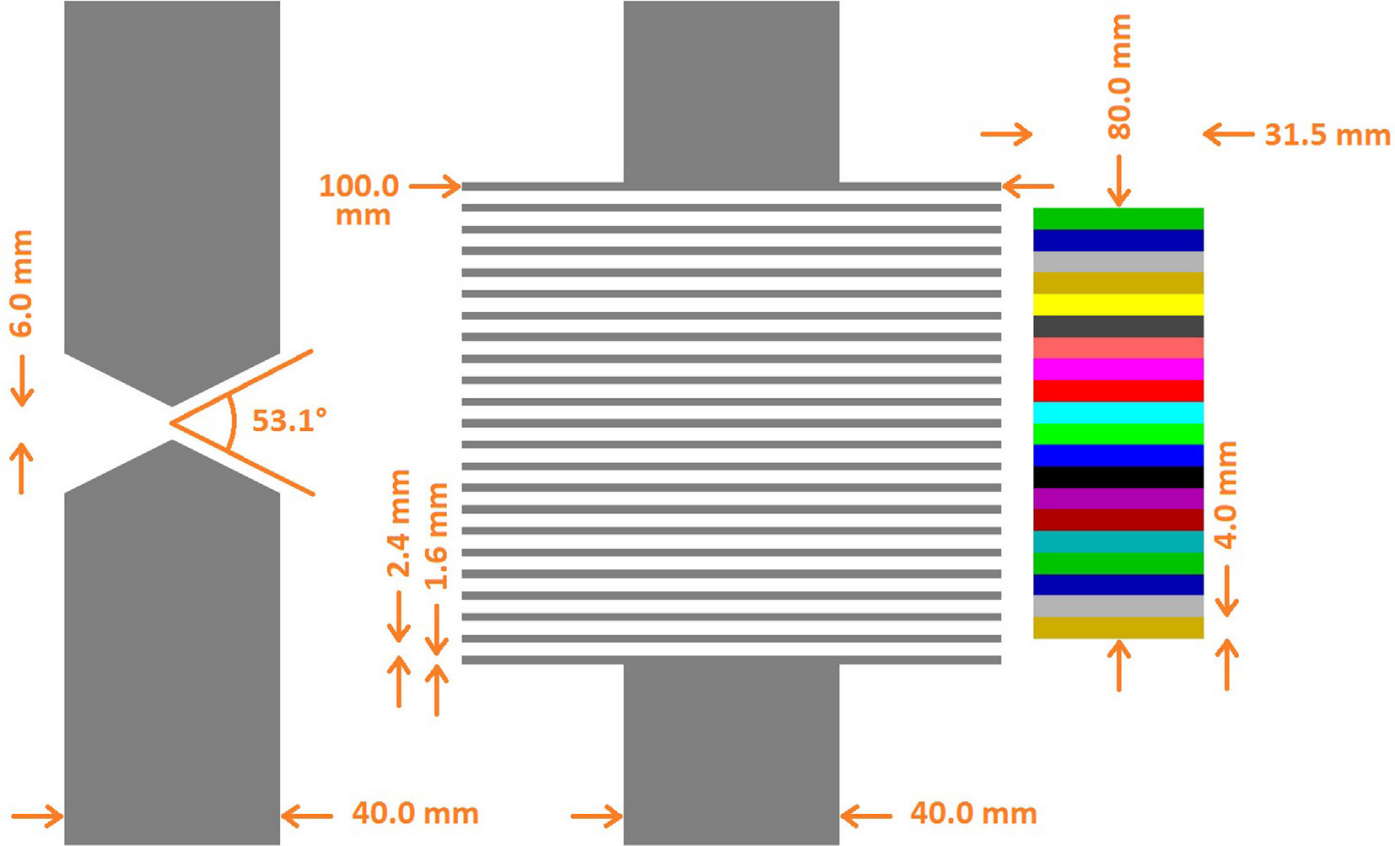
**Superimposed collimator geometries**. The KES collimator (left) is superimposed with the MPS collimator (center) and the crystals (right). All distances with respect to the crystals are conserved. In the real prototype version (cf. Figure 2) used for beam tests, the parallel apertures of the MPS collimator are 0.1 mm wider (2.5 instead of 2.4 mm) in order to preserve alignment in presence of the 0.1 mm absorber sheets inserted in between the crystal slabs of the real camera device.

Both collimators are schemed in Figure 1. The KES collimator has a single 6 mm and 53.1° [=2*acot(2)] aperture. The MPS collimator has parallel apertures of 2.4 mm gap separated by tungsten alloy sheets that are 1.6 mm thick and 100 mm deep, matching the 4 mm segmentation of the camera and resulting in a fill factor of 40% which, combined with the 100 mm depth, equals the 40 mm thickness of the KES collimator in terms of attenuation efficiency. In line with their optimizations, the KES collimator is used in a 5:4 magnification ratio corresponding to 10 cm Field-Of-View (FOV) along beam axis, while the MPS collimator positioned right against the camera in a 1:1 magnification ratio corresponding to 8 cm FOV.

Roellinghoff (16) showed that a comparison of KES and MPS collimators, in the ideal conditions of a MPS collimator with infinitely thin septa and a KES collimator of which the solid angle variation along the FOV would be neglected, can result in fully identical Bragg peak retrieval precisions and spatial resolutions upon relevant scaling of the dimensions. Simulations with GATE then brought confirmation that this result can reasonably hold when considering realistic designs. Similar performances can be targeted in a general context. In the present, practical context, we deviated in at least two notable ways from conditions of equal performance. First, a MPS collimator to be compared to the present KES one would preferably involve a larger pitch than the present 4 mm one (actually a 12 mm one) to compromise spatial resolution at the benefit of detection efficiency, which would have required an alternative manufacturing of the present camera system. Second, we did not impose coherent scaling of the distances between beam axis, collimator and crystals for both collimators. We instead decided to only impose an identical distance between beam axis and the collimator entrance face (actually 200 mm) in order to reflect a practical constraint of positioning the camera as close as possible to beam axis while avoiding collision with the patient. Beyond this collimator entrance face constraint, crystals were independently positioned at optimal distances, which results in a closer distance to beam axis (and subsequently in a favored detection efficiency) for the MPS setup over the KES one. The overall balance of these two deviations from conditions of equal performance is a better detection efficiency for the KES collimator and a better spatial resolution for the MPS one.

### Proton Beam Tests

Measurements were performed in October 2013 at the West German Proton Therapy Centre Essen (WPE) with a proton beam delivered by an IBA C230 isochronous cyclotron in a treatment room equipped with a PBS-dedicated nozzle. All acquisitions were performed for 10 s from a single spot at isocenter delivered along the axis of a PMMA target that is 7.5 cm in radius. Delivered proton charge was integrated by an electrometer connected to an ionization chamber intercepting the whole section of the pencil beam inside the nozzle. With the beam already on, the 10 s charge integration was synchronized manually with each camera acquisition. This was observed to result in a maximum error of 3% in charge collection over six repetitions of a same acquisition. Accurate absolute calibration was not required for our comparative evaluation and the ionization chamber was therefore not calibrated for the temperature and pressure of the day, so that the absolute calibration cannot be assumed to be at the 1% level. The energy calibration of the camera was performed by combining the characteristic gamma rays identified from a spectrum acquisition of the prompt gammas emitted by a water target during proton irradiation and a spectrum acquisition of the gammas resulting from the decays of Na-24 produced by the previous proton irradiation of an aluminum target.

**Figure 2:**
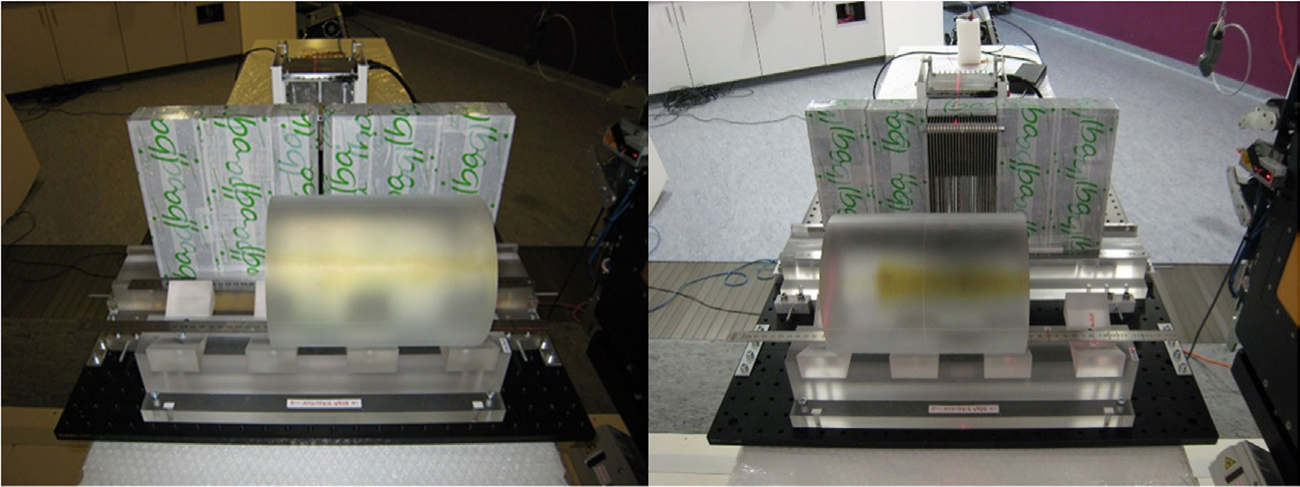
**Experimental prompt gamma camera setups**. The KES collimator setup is pictured on the left and the MPS collimator on the right.

Both collimator setups are pictured in Figure 2. For direct comparability, the two collimators were positioned with their entrance face at 200 mm from beam axis. As a result, the center of the KES aperture was 220 mm from beam axis and the center of the crystals was 176 mm from the center of the KES aperture, while the center of the MPS collimator was 250 mm from beam axis and the crystals were right behind.

Acquisitions were recorded with each setup at 100, 160, and 230 MeV to cover the clinical range, first with the center of the FOV aligned with the expected range in PMMA (6.7 cm at 100 MeV, 15.2 cm at 160 MeV, and 28.4 cm at 230 MeV) and second with the center of the FOV aligned with the entrance point of the beam inside the target. The cylindrical PMMA target was 20 cm along beam axis at 100 and 160 MeV and 40 cm at 230 MeV. Acquisitions were recorded with different energy windows and only the ones with the window 3–6 MeV are presented here as they were assessed to result in the preferred compromise between count rate, robust calibration and, most importantly, falloff retrieval precision over the different beam energies. All acquisitions were recorded at beam current values within the clinical range: ~1 nA at 100 MeV, 2 nA at 160 MeV, and 4 nA at 230 MeV.

### Performance Evaluation

The performance of either collimator setup in each acquisition was rated by applying the approach of Roellinghoff et al. (18) as described in Perali et al. (11). Starting from the very high statistic detection profile of the 10 s acquisition, corresponding to the order of 10^11^ protons, 1000 profiles were sampled for three different numbers of protons (1E8, 3E8, and 1E9 protons) and were then matched with the original very high statistic profile (as if it were the result of the expected signal computation model once perfectly calibrated) in order to estimate the intrinsic falloff retrieval precision. The lateral shift between each low-statistic sample profile and its high-statistic original profile is determined as the one minimizing the root-squared difference between the two profiles from all tested shifts between −20 mm and +20 mm by steps of 0.25 mm. This lateral shift should here equal 0 in case of exact retrieval and the average error over the 1000 sample profiles delivers a reliable indication of the intrinsic quality of the detection profile generated by either collimator. The larger the amplitude of the prompt gamma signal detected thanks to a good detection efficiency and/or the sharper the edges of the detected falloff thanks a good spatial resolution, the better the falloff retrieval precision. Roellinghoff et al. (18) showed that the falloff retrieval precision is inversely proportional to the square root of the number of protons, so that they exhibit a linear relation in a log–log plot. For each acquisition of either collimator setup, this linear relation was interpolated so as to determine the number of protons corresponding to a 2 sigma precision of 4 mm, which, in line with Perali et al. (11), was arbitrarily chosen as reference for our study.

In order to improve the falloff retrieval precision, all profiles where applied a Gaussian smoothing with a Full Width At Half Maximum (FWHM) equal to that of the impulse response of the collimator as determined from simulations with Monte Carlo code MCNPX version 2.5.0. This smoothing advantageously attenuates the spatial frequencies that are too high to result from the collimator projection and that essentially correspond to slab-to-slab variations in the number of counts due to statistical fluctuations and, to a lesser extent, to the lack of uniformity resulting from uncertainties on the individual energy calibration of each slab and, in case of the MPS collimator, from the uncertainty (±0.1 mm) on the thickness of the tungsten alloy sheets causing some of the parallel apertures to be slightly wider or narrower.

## Results

**Figure 3:**
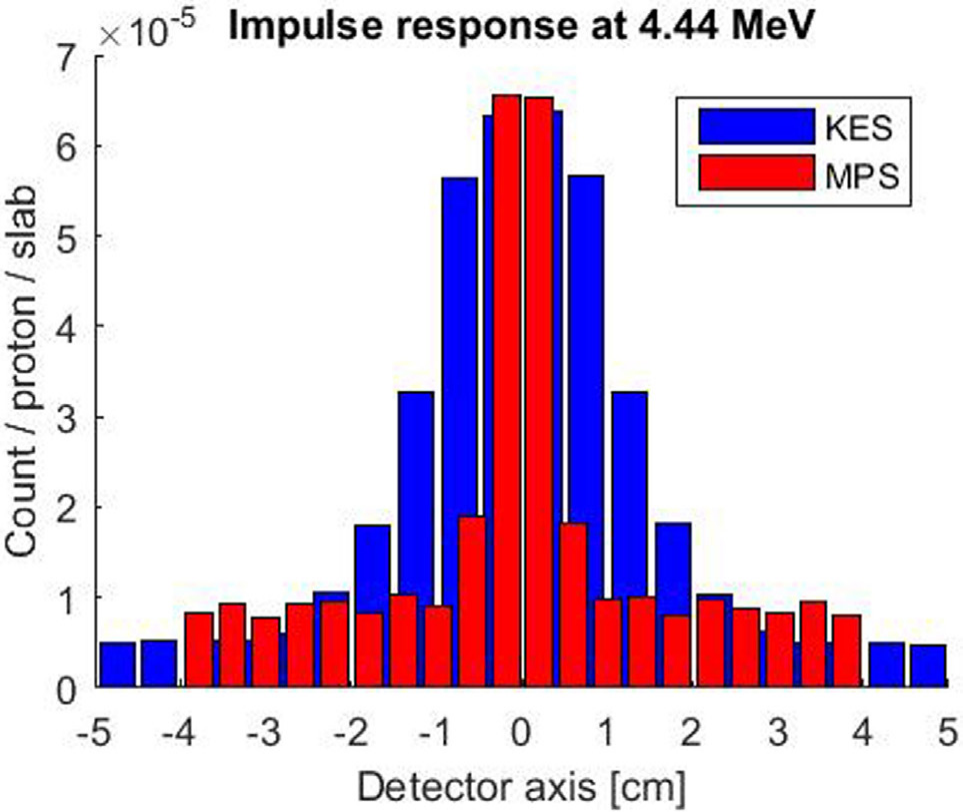
**Simulated impulse response of both collimator setups for a 4.44 MeV point source along beam axis at the center of the FOV**. The KES collimator response is plotted in blue and the MPS one in red.

The simulated impulse response of both collimator setups is compared in Figure 3. A 4.44 MeV gamma point source was considered for this evaluation as it is the most intense characteristic prompt gamma ray resulting from the irradiation of carbon and oxygen at the center of our 3–6 MeV window. The MPS collimator exhibits a thrice better spatial resolution with 7 mm FWHM versus 22 mm for the KES one. The KES collimator is scoring more signal with poorer spatial resolution and records a slightly lower background of uncorrelated signal. In the KES configuration, the crystals are further both from the collimator and the beam axis, which reduces the detection efficiency of gammas that, with or without scattering, succeed in emerging from the 40 mm tungsten thickness.

**Figure 4:**
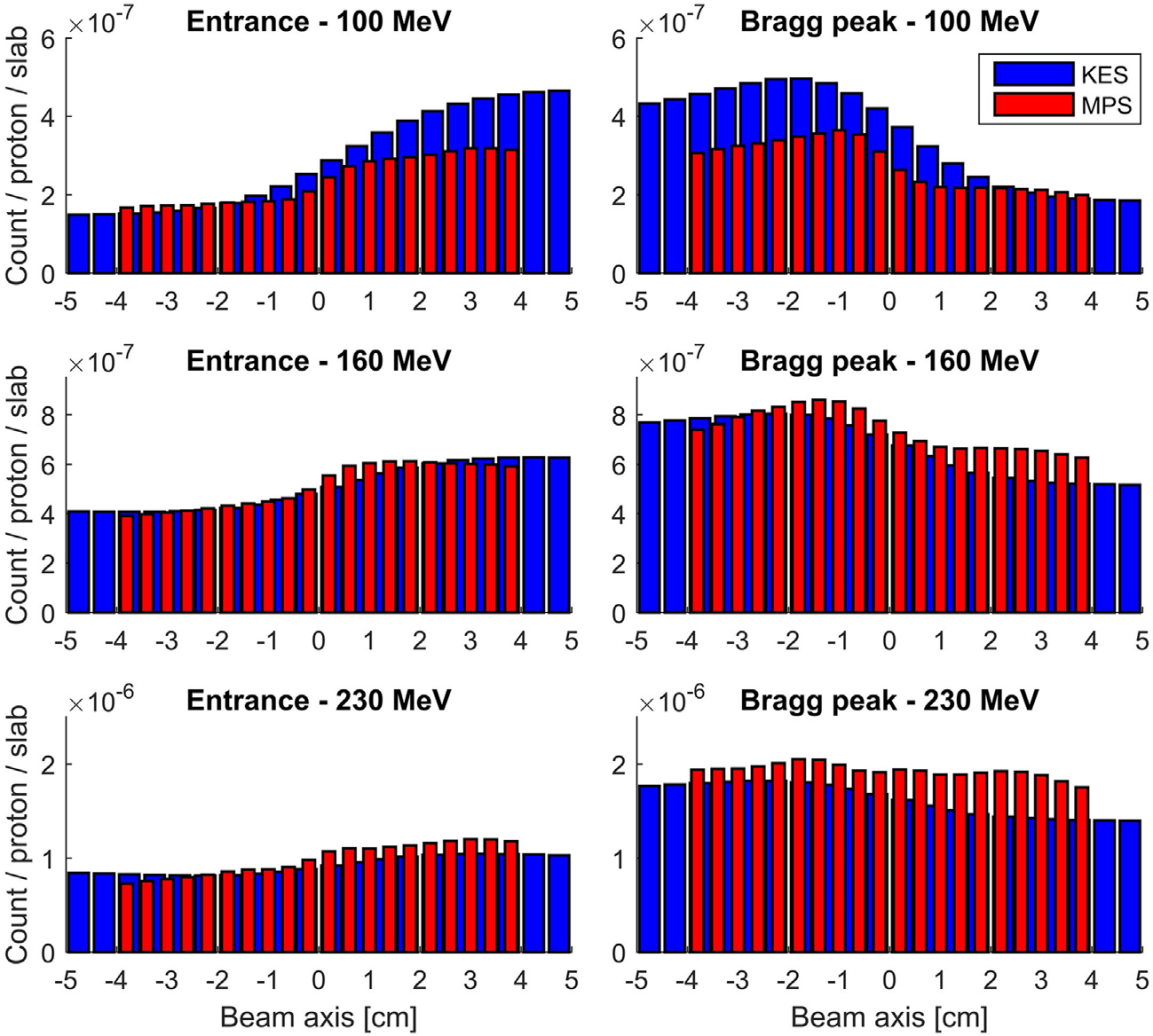
**Measured prompt gamma profiles with a 3–6 MeV energy window**. All acquisitions were performed for 10 s at clinical beam currents between 1.1 and 4.7 nA, corresponding to numbers of protons incident on the target between 7E10 and 3E11. The KES collimator response is plotted in red and the MPS one in blue.

The detection profiles recorded by both collimators at 100, 160, and 230 MeV, when imaging the Bragg peak as well as when imaging the entrance of the beam in the target, are compared in Figure 4. The KES and MPS acquisitions were applied a 22 and 7 mm FWHM Gaussian smoothing, respectively.

The performance of each acquisition in Figure 3 is rated in Table 1 in terms of computed number of protons (in units of 1E8 protons) necessary to reach a 2 sigma precision of 4 mm on range estimation. Each value is the average over three computations with different seed numbers to the random number generator that is used to generate the sample profiles from the measured one according to a Poisson process. The relative SD of the three computations ranged from 2 to 6%.

**Table 1:** **Computed number of protons (in units of 1E8 protons) necessary to reach a 2 sigma precision of 4 mm on range estimation for the detection efficiency of the full camera**.

	Entrance		Bragg peak	
	KES	MPS	KES	MPS
100 MeV	0.82	1.42	0.35	0.65
160 MeV	2.92	1.87	1.19	1.71
230 MeV	3.15	1.62	2.01	4.44

*Each value is the mean value of three computations with different seeds to the random number generator. The relative SD of the three computations ranged from 2 to 6%*.

For all acquisitions in Table 1, the number of protons to reach a 2 sigma precision of 4 mm on Bragg peak (or entrance) falloff retrieval is in the order of 10^8^ protons, showing that statistically meaningful feedback is possible on a single spot basis for the few highest weighted spots of the order of 10^8^ protons close to the target distal edge. On the other hand, neighbor spot aggregation will be necessary for the majority of spots of the order of 10^7^ protons, and no statistically meaningful information can result from the proximal lowest weighted spots of the order of 10^6^ protons.

A first remark on these results is that the performance criterion of a 2 sigma precision of 4 mm was considered here because it applies identically to the retrieval of both the Bragg peak and the entrance point of each pencil beam for direct comparison and is independent of the choice of any distal margin recipe. As a consequence, this criterion fails to reflect the fact that achieving a 2 sigma precision of 4 mm at 230 MeV is clinically much more valuable than achieving it at 100 MeV in terms of margin reduction. If we arbitrarily assume a distal margin recipe of 3.5% + 2 mm based on 1.5 sigma (19), the distal margin in our PMMA target would be 4 mm at 100 MeV and 12 mm at 230 MeV at the 1.5 sigma level.

A second remark is that the performance criterion of a 2 sigma precision of 4 mm is an arbitrary choice applied to the context of this collimator comparison and is not a lower bound on the precision achievable by either collimator in any context. Increasing the number of protons considered, positioning the collimator closer to beam axis, reducing beam energy, and increasing the oxygen to carbon composition ratio in the target are all factors that, alone or combined, would cause a better precision in other contexts.

A third remark is that, at the date of these measurements, only one of the two rows of 20 LYSO slabs was mounted on camera, so that the detection efficiency reported in Figures 3 and 4 is exactly half that of the full camera. For a more meaningful rating of the performance of the camera, we assumed the double detection efficiency of the full camera in the performance values further reported in Table 1.

## Discussion

**Figure 5:**
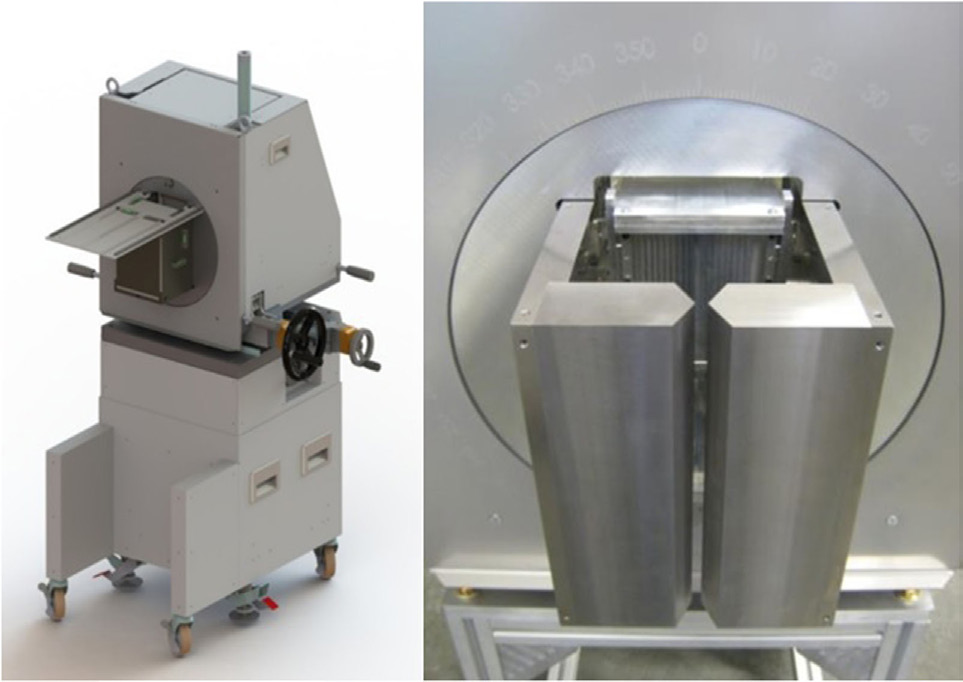
**Prompt gamma camera prototype trolley positioning system**. The complete trolley is drawn on the left and the real KES collimator is pictured on the right.

Performance values in Table 1 reveal two trends. First, whatever the collimator, increasing beam energy reduces the performance. This was fully expected both from simulations and past measurements by Min et al. (5) for the MPS collimator and Smeets et al. (12) for the KES one. Second, the Bragg Peak retrieval performance of the KES collimator is better than the MPS one in combination with the camera device under consideration. Roughly twice less protons are needed by the KES to reach a given precision. This result was the very focus of the present study, and the KES design was therefore selected to equip the present prompt gamma camera device for further assessment of its performance during clinical treatment delivery as illustrated in Figure 5. The very first prompt gamma acquisition of a patient treatment was recently performed with it by Richter et al. (20) at the Universitäts Protonen Therapie Dresden at OncoRay.

Beyond these first observations, performances values in Table 1 highlight another interesting finding. For the KES collimator, the performance in retrieving the entrance position also degrades when increasing beam energy and, whatever the energy, the performance in retrieving the entrance position, is always poorer than the performance in retrieving the Bragg peak position. This was already demonstrated in Perali et al. (11). In contrast, the MPS collimator succeeds in maintaining a valuable and stable performance at all beam energies for the entrance point. As a result, at 160 and 230 MeV, the MPS collimator not only exhibits better performance for the entrance than for the Bragg peak but also achieves better performance for the entrance than the KES collimator.

The origin of these different behaviors at entrance and Bragg peak lies in the anisotropy of proton-induced neutron emissions that generate most of the measured background signal at high beam energies. When either collimator is aligned at the entrance face of the target, the measured neutron background is not uniform but sloped because proton-induced neutron emissions are forward-peaked and, in contrast to the Bragg peak depth, there are at the entrance depth no neutrons generated downstream to compensate the anisotropy of those emitted beyond the entrance depth. Both collimators are inefficient at collimating those neutrons. In the case of the KES collimator, the neutron background at target entrance has an opposite slope to that of the reversed 1D projection of the correlated prompt gamma, whereas both components add up slopes of equal sign in case of the MPS collimator. As a consequence, when beam energy increases, the slope of the neutron signal gradually cancels that of the prompt gamma signal projected by the KES collimator, whereas it adds a positive contribution to that of the prompt gamma signal projected by MPS collimator and roughly compensates for the reduced prompt gamma emission at entrance by higher energy protons so as to maintain a rather stable performance whatever the beam energy.

The conclusion of the present study is that KES collimator proved better for Bragg peak depth retrieval, whereas the MPS collimator proved better for entrance depth retrieval. On the one hand, it is unfortunate as it would have been more convenient to benefit from the best performance for both extremities of the proton range with one single design. On the other hand, it is fortunate that the MPS collimator is the one achieving the best performance for the entrance position as it is also the one the FOV of which can most straightforwardly be increased in order to image the whole proton track without compromising the uniformity. The compact prototype in Figure 5 was built with a KES collimator for clinical evaluation as it is meant for the measurement of the Bragg peak depth with its 10 cm FOV. Measuring the entrance point by PGI implies a significant increase in cost, weight and footprint of the camera in order to cover proton ranges up to 32 cm in patients that need be evaluated in terms of clinical value. Further investigations will assess the combination of the Bragg peak image by the camera with other imaging modalities (X-ray shots, CBCT, and/or optical tracking) that have the potential to advantageously substitute the PGI acquisition of the entrance depth.

Finally, two limitations to the generality of the results of the present study should be underlined. First, the 4 mm segmentation of the camera system (resulting from an optimization of the tradeoff between the detection efficiency, the count rate and the number of channels and photodetectors) is not optimal for an MPS collimator that tends to favor larger pitches (16, 21) whereas the KES collimator performance is less sensitive to any variation of the crystal segmentation (12). Second, the MPS collimator was here suffering from a higher level of background when imaging the Bragg peak at high beam energies and it might therefore be anticipated that the addition of any background reduction method (at the cost of an increase in the complexity of the camera design), for example by means of a TOF discrimination technique (22), would benefit more to the MPS than to the KES collimator. A comparison of the performance of KES and MPS collimators in the context of a camera with a different segmentation and/or featuring any additional background discrimination technique (and thereby relying on a different tradeoff between cost, performance, and complexity) may lead to different conclusions.

## Author Contributions

Collimator design simulations and manufacturing: FR, JS, NF, ET, and DP. Camera design, manufacturing and calibration: IP, AC, and CF. Experiment preparation: FR, JS, GJ, IP, AC, CF, and DP. Experiment conduct: JS, FR, GJ, IP, AC, and DP. Data analysis methodology: FR, GJ, JS, NF, and ET. Data analysis software: GJ. Data analysis conduct and result redaction: JS. Result discussion and manuscript review: FR, GJ, IP, AC, CF, NF, ET, and DP.

## Conflict of Interest Statement

Part of the authors and their institutions have filed patent applications relevant to this work. The authors declare that the research was conducted in the absence of any commercial or financial relationships that could be construed as a potential conflict of interest.
